# Supplementary material for: Socio-economic and environmental factors affecting breastfeeding and complementary feeding practices among Batwa and Bakiga communities in south-western Uganda
Source: PLOS Glob Public Health. 2022 Mar 9;2(3):e0000144. doi: 10.1371/journal.pgph.0000144 (PMC10021580; doi:10.1371/journal.pgph.0000144)
Supplement: S1 Text — (DOCX) [file pgph.0000144.s002.docx]

**S1 Text**

**Individual interviews guide questions**

1. What is your ethnicity?
2. What is your age? (maternal age)
3. What age is your baby?
4. Is this your first baby?
5. If no, how many other babies have you had?
6. Do you have any twins?
7. What is your age?
8. What do you do during the day to find food or earn a wage?
9. Do you bring your child with you everywhere you go (e.g. work place, farm, etc.)?
10. Are you allowed to breastfeed your child when working? Do you breastfeed him/her at work?
11. Do you receive food from any NGOs/government?
12. Does your household own any animals? Any lands?
13. Do you have a toilet/latrine?
14. Do you have access to soap?
15. Do you have running water?
16. When do you wash your hands?
17. Where was your baby born (Hospital/Home/health centre)?
18. How was your delivery (vaginal/ c-section)?
19. Any complications during the delivery? If so, explain it please.
20. Have you ever breastfed this baby? For how long?
21. Were you and/or your baby sick at birth?
22. Was your baby sick in the first 6 months of life?
23. If s/he was sick, what did she/he have, if you know? (For example: fever, diarrhoea, vomiting, respiratory problems, skin problems, oedema, very thin, big belly (signs for malnutrition), worms, eyes problems, constipation, urinary problems, malaria…)
