# Supplementary material for: Socio-economic and environmental factors affecting breastfeeding and complementary feeding practices among Batwa and Bakiga communities in south-western Uganda
Source: PLOS Glob Public Health. 2022 Mar 9;2(3):e0000144. doi: 10.1371/journal.pgph.0000144 (PMC10021580; doi:10.1371/journal.pgph.0000144)
Supplement: S1A Text — (DOCX) [file pgph.0000144.s003.docx]

**S1A Text**

Translation in Rukiga language

**Ebibuuzo byaburimuntu**

1. Ori owarurimikyi?
2. Oyiine emyaaka engahi? (emyaaka eyabakazi)
3. Omwaana wawe ayine emyaka engahe?
4. Ogu nomwana wawe wokubanza?
5. Ku arabe ateri wokubanza, abandi abaana oyiine bangahi?
6. Oyine a barongo?
7. Oyiine emyaka engahe?
8. Nokorakyi omwihangwe kugira ingu obone ebyokudya ninga omushaara?
9. Nogenda nomwana waawe burihamwe ahorikuza? (ekyokureberaha ahorikukora, omumusiri nahandi nahandi )
10. Noyikirizibwa kwonsa omwana waawe waaba orikukora? Noyonsa waaba okozire?
11. No yakyira emere kurunga omubitongore ninga omugavumenti?
12. Eka yaawe eyine etuno dyoona? Kandi itaaka?
13. Oyiine kamiina?
14. Noboona aha sabuuni?
15. Oyiine amaizi garwesindika?
16. No naaba omungaro dyari?
17. Okazariira omwaana waawe inkahe (omwigwariro erihango?
18. Okazara ota ( okazara je/ bakakushemwza) ?
19. Hariho ebizibu ebiwaatungire orikuzaara ? kukyiraabe kyiriho kyishoborore?
20. Waronkyizeho ogumwaana? Kumara obwiire burikwinganakyi?
21. Iwe ninga omwana waawe mukaba mugwiire obuwazaara?
22. Omwaana waawe akagwaraho omunyezi mukaaga (6) yokubaanza?
23. Ku arabe yagwiire akaba agwiire kyi ku orabe nokyimanya ? (ekyokurebare: omuriro, ekyirukano, okutanaka, okwiisa kubi, oburwire bwomubiiri, okuzimba, okukooha, mutuku, enjoka, ebizibu byamaisho, okunyempata, obuzibu omukushesha, omushiaja rwesiiri….)
