# Supplementary material for: Socio-economic and environmental factors affecting breastfeeding and complementary feeding practices among Batwa and Bakiga communities in south-western Uganda
Source: PLOS Glob Public Health. 2022 Mar 9;2(3):e0000144. doi: 10.1371/journal.pgph.0000144 (PMC10021580; doi:10.1371/journal.pgph.0000144)
Supplement: S2 Text — (DOCX) [file pgph.0000144.s004.docx]

**S3 Text**

**Focus group discussions guide questions**

The questions in bold are the main ones, while the others can be asked (if necessary) to define better the main question.

1. **Where do mothers get information on breastfeeding? And on complementary feeding?** Who helps mothers to learn about breastfeeding? And weaning? Do you know if there are specific courses on breastfeeding from the hospital or any other organization (e.g. NGOs) offered to any woman during pregnancy? And for weaning? Which are the courses? What do you know about HIV and breastfeeding?
2. **What determines whether a mother breastfeeds or not?** What are the reasons women in your community decide to breastfeed or not? Who? How soon after birth would a woman in your community breastfeed the baby? Is there anyone else breastfeeding the baby instead of the mother? Who?
3. **Does weather or environment affect breastfeeding or weaning?** Are there specific seasons in the year when breastfeeding or weaning are easier? Why? Are the foods used for complementary feeding different from years ago? What do you use instead now? Why? In your opinion, does this change have an impact on child growth? Why?
4. **Do you ever have problems with breastfeeding? And with complementary feeding?** Do you think some women cannot breastfeed their babies? Why? Can breastmilk not be enough for the baby? Why? If you feel you do not have enough breastmilk, what can you do to produce more? What are the barriers that could limit breastfeeding (e.g. do mothers eat enough?)? Who helps the mother during breastfeeding?
5. **Is there anything that help or would help mothers with breastfeeding?** Is there anything that would help mothers breastfeed longer?
6. **When do mothers usually wean their babies?** Does a baby usually receive any other liquids (e.g. water, milk, etc.) other than breastmilk? Which ones? Is there any special food eaten during the breastfeeding period? If so, what foods? When does usually a baby start to eat solid food? What are the first solid foods? Is there anyone else feeding the baby instead of the mother? Who? What are the barriers that could limit an appropriate weaning? Is there anything that would help mothers in having more knowledge on complementary feeding/weaning practices?
7. **Do you worry about your infants being malnourished?** Do you think there are malnourished infants in your community? How do you recognise them? What should the mother do if the child is malnourished?
