# Supplementary material for: Socio-economic and environmental factors affecting breastfeeding and complementary feeding practices among Batwa and Bakiga communities in south-western Uganda
Source: PLOS Glob Public Health. 2022 Mar 9;2(3):e0000144. doi: 10.1371/journal.pgph.0000144 (PMC10021580; doi:10.1371/journal.pgph.0000144)
Supplement: S2A Text — (DOCX) [file pgph.0000144.s005.docx]

**S4A Text**

**Ebibuzo byabetarine**

Ebibuzo ebikwasiire nibyobyamaani, ebiindi no basa kubibuza kyayetangisa kugira ingu oshoborore ekyibuza ekyamaani.

1. **Abaakazi nibiiyahe amakuru agakwasire aha kwonsa? Hamwe na nokwongera ebyokudya omwaana okyayonka?**

Nooha orikuyaamba abaakazi kweega ebyokonsa? Nanakukyusa? Nokyiimanya haaba hariiho omushomo rwokwonsa ahirwariro ninga ebiindi ebitongore? Ebibaraha omukaazi weena yaaba ayiine enda? Nanokukyusa? Emishimo neeha? Nomanyakyi aha kaakoko kasiirimu hamwe no kwonsa?

1. **Ekirasharamu yaaba omukazi ayonsa ninga atayonsa nikyi?** Enshonga omukyaaro kyaawe ezikaretera omukaazi yayonsa ningashi atayonsa niiziha? Nooha? Nibwirekyi obyomukaazi arikwonkyenzamu yamara kuzaara? Hariho ondijo orikwonsa omwaana Atari nyina? Nooha?
2. **Empinduka yobwire ninga ebyobuhangwa nibiteganisa bita okwonsa no kukyusa?** Hariho obwire obwokonsa nokikyusa birikwanguha? Ahabwakyi? Ebyokudya ebimurikuha abaana kwonjera ahamashereka bihindukyire kuraga ahanyaka ewiire? Haati nokozesakyi hati? Ahabwakyi? Iweomukureba kwawe egimpinduka eyina okwerikuteganisa aha kukura kwo omwaana? Ahabwakyi?
3. **Hariho obworikugire ebizibu nokwonsa? Hamwe nokwonjera ebyokudya ahamashereka?** Notekateka hariho abakazi batarikyonsa abaana baabo? Ahabwakyi? Amashereka nigabaasa kuba makye aha mwaana? Ahabwakyi?Wahurira otine mashereka nokorakyi kugira ingu ogire amashereka mingi?. Nibintukyi ebirikukyendeza amashereka? ( ekyokureberaho abakaazi tibakadya ebirikumara?) Noha orikuyamba abakazi baaba nibonsa?
4. **Hariho ekyintukyona ekyirikuyamba abakazi baba nibonsa?** Hariho ekyiri kuyanba abakazi kwonsa kumara obwire buringwa?
5. **Abakazi nibakyira kukyusa abaana baabo dyari?**omwaana nakyira kubona ebindi ebyokunya ( amaizi, amate, nanabindi….) gatari mashereka? Nibiha? Hariho ekyokudya kyomutano ekyabakazi barikudya baba nibonsa?byaba biriho nibiha? Omwana nakyira kutadika kudya dyari ebyokudya bigumire? Nibyokudya kyi bigumire? Hariho ondijo orikurisa omwaana Atari nyina? Noha? Nibintu kyi ebirikuteganisa okukyusa gye? Hariho ekyintu kyoona ekyirikuhereza omukazi obwengye ahakwonjera ebyokudya omwaana akyayonsa ninga okukyusa?
6. **Notiina omwaana wawe yagira mutuku?** Notekateka hariho omwaana arwire mutuku omukyaro kyawe? Nobamanyirahe? Omukazi ashemerire kukorakyi omwaana weye yagira mutuku?
