# Supplementary material for: Socio-economic and environmental factors affecting breastfeeding and complementary feeding practices among Batwa and Bakiga communities in south-western Uganda
Source: PLOS Glob Public Health. 2022 Mar 9;2(3):e0000144. doi: 10.1371/journal.pgph.0000144 (PMC10021580; doi:10.1371/journal.pgph.0000144)
Supplement: Attachment — Submitted filename: Response to reviewers_02122021.docx [file pgph.0000144.s007.docx]

| We are very thankful for your suggestions and contributions. We have incorporated all of your suggestions into this revision. | |
| --- | --- |
| Response to reviewer comments and revisions  December 2nd, 2021 | |
| Reviewer comments | Author comments/response |
| **Reviewer #1:** | **Author comments/response** |
| 1) Line 68: ………. “and food behaviour among Indigenous families due to changing in socio-environmental”; replace the word “changing” with “changes”. | Thank you for your feedback.  This revision has now been made. |
| 2) Line 174: “Fig.2: In the map we represented the ten Batwa settlements that participating in the research; there is….” replace the word “participating” with “participated”. | This revision has now been made. |
| 3) Line 183: “Kihembe/Kengoma cell), 2) two settlements closed to the forest and located…..” replace the word “closed” with “close”. | This revision has now been made. |
| 4) Line 202: “singular) female researcher. The FGDs and the individual interviews were conducted and audio-recorded in….” – write the FGDs in long form since it is the first time it is used in the main text and delete “The”. Therefore, line 202 will read: “singular) female researcher. Focus group discussions (FGDs) and the individual interviews were conducted and audio-recorded in….”. | This revision has now been made. |
| 5) Line 206: “We created an interview guide with open questions for the focus group discussion (Appendix 1).” – replace focus group discussion with FGD. | This revision has now been made. |
| 6) Line 207: “followed the themes from the Optimal Infant and Young Child Feeding guidelines,” – replace Infant and Young Child Feeding with IYCF, because it has been written in long form in line 53. | This revision has now been made. |
| 7) Lines 254-256: “Mothers of all children older than 7 days reported that their child had experienced one or more episodes of illness in the first 6 months of life that had had a negative impact on breastfeeding.” It is not clear if the authors mean 7 days OR 7 months. I suggest to authors to re-check on this. | We confirm that we are referring to children older than 7 days. |
| 8) Lines 393-396: “Information on child feeding is provided to mothers by the hospital or NGOs (Tab. 4; the main topics covered in these sessions includes breastfeeding practices, complementary foods and malnutrition, importance of giving birth at the hospital and vaccinating children, family planning, and HIV testing before delivery.” I suggest to authors to close a bracket after 4, i.e., (Table 4); the main topics……… | The reference has been added. |
| 9) Line 407: I suggest to bold the subheading “Cultural beliefs” for consistency. | This revision has now been made. |
| 10) Lines 475-477: “Participants did not limit their recommendations to improved food security only, however, and recognized barriers at opportunities across levels identified in our framework (Figure 1).” This sentence is not clear in its last part underlined. I suggest to the authors to consider rephrasing it to read as follows: “Participants did not limit their recommendations to improved food security only, but they recognized barriers and opportunities across levels identified in our framework (Figure 1).” | This revision has now been made. |
| 11) Line 503: “consequences of alcoholism in the community were identified as key needs). Indeed, research has….” – the bracket after “needs” is not needed; just delete it. | This revision has now been made. |
| 12) Line 517: “However, participants also highlighted the important role of traditional birth attendants (TBAs) to provide….” - delete “traditional birth attendants” and use only TBAs without brackets because it has already been written in long form and abbreviation in Line 409. | This revision has now been made. |
| 13) Line 535: “and maternal and neonatal health. For example, Bryson et al. (25) described the linkbetween pregnancy” – separate the word “linkbetween” to be two words. | This revision has now been made. |
| 14) Line 566: “and complementary feeding practices in the area may involve fathers, traditional birth attendants and health” – replace “traditional birth attendants” with TBAs | This revision has now been made. |
| 15) Line 568: “practices among men. Indeed, the focus on infant and young child feeding, and in particular on the first 1000” – replace “infant and young child feeding” with IYCF. | This revision has now been made. |
| 16) Line 573: “participated to the interviews and focus group discussions, and gave recommendations to improve child feeding” – replace “focus group discussions” with FGDs. | This revision has now been made. |
| 17) Figure 2 (page 36): “Fig.2: In the map we represented the ten Batwa settlements that participating in the research; there is a correspondent Bakiga settlement for each Batwa settlement. In our study, we involved……….” – replace “participating” with “participated”. | This revision has now been made. |
| **Reviewer #2:** | **Author comments/response** |
| 1) there seems to be a disconnect between the data presented on Table 1 on birth interval and the claims from the FDG that Batwa women get pregnant fast on line 370 kindly qualify that and probably link that to teen pregnancies. | Thank you for this comment. We have clarified in the Results section that teenagers and young women are more likely to get pregnant faster (<24 months from the previous birth) according to the Batwa participants (line 373-375):  *‘According to the participants, young women and teenagers are in fact more likely to have their next child within 23 months due to lack of information and lower use of contraception.’* |
| 2) The data provided seemed to have only one set of twins yet your findings though resonable provide strong claims on transistions for weaning practices. I would suggest that you update this to also include competition of food resources for young children with many siblings <5 years to strengthen the claim | Thank you for the suggestion. We have added this in line 309-312 of the text:  *‘Participants added that appropriate complementary feeding practices get more complicated to follow in case of twins or multiple children under 5 years of age as it is difficult to provide sufficient foods to satisfy their nutritional needs. Also, they argued that is harder to produce enough milk for two or more children at the same time, therefore weaning usually starts before 4 months (Bakiga FGD).’* |
| 3) I suggest adding a short paragraph about recommendations after the results and move table 3 from the discussion section below the new paragraph. Also improving food availability and accessibility is a very generic recommendation in this case, instead when writing the paragraph mention that recommendations targeting improvement of food availability and accessibility were identified as summarised on Table 3. | Thank you for the suggestion. We made the change in the text (line 479 to 483):  *Additionally, we synthetisized recommendations to improve breastfeeding and complementary feeding practices among Batwa and Bakiga communities (Table 3). These included suggestions to enhance the food environment, such as food availability and accessibility, health services, and community and medical support as summarised in Table 3; they recognized, also, barriers and opportunities across levels and themes identified in our framework (Figure 1).* |
| 4) On the table 3, levels are confusing, instead replace with themes that emerge from your study such as support etc. | We modified the table as requested by the reviewer (see Table 3). |
| 5) I would suggest adding an additional recommendation on climatic changes if in this communities sustainable farming such as irrigation and production of drought resistance crops as well as improving drainage through contours are feasible to counter effects of floods and droughts. Kindly review available literature to support this argument. | Thank you for the suggestion. We added a new recommendation (recommendation 8) to Table 3: the promotion of sustainable agriculture to better adapt to extreme climatic events (especially on droughts and floods) and increase food production and availability. Also, we included the following text to the Discussion section (line 562-572):  *‘Research has demonstrated that promoting sustainable agriculture is key to maintain and/or increase food security in a changing climate (70). Sustainable agriculture has been identified as an important climate response with the potential for double benefits: potential reduction in emissions and adaptation to the implications of climate change for food security (71). Common principles of sustainable farming include intercropping, high plant diversity and seed recycling (72). Efficient adaptation strategies to climate change stressors on agricultural productivity will be important in underpinning nutritional outcomes for Batwa and Bakiga mothers and children during the breastfeeding and complementary feeding period.* *However, transitioning to climate-smart or sustainable agriculture in many cases requires a strong knowledge base, financial resources, and adequate policies; for populations in extreme poverty such as the Batwa – where baseline development remains severely inadequate − transitions to sustainable pathways are unlikely to be feasible without substantial and targetted support.(73)’.* |
| 6) On discussion kindly expand paragraph 1 to showcase what are the deviations and similarities of factors identified from studies identified by UDHS to support your claim on focussing on indigenous communities whose issues may be overlooked from UDHS findings. Also define UDHS in full not every reader can relate that you are talking of Uganda demographic Health Surveys. | We have added the following text to the manuscript (from line 470-476) and we have defined UDHS in full:  *‘Other studies in Uganda have only quantitatively investigated factors affecting complementary feeding by using Uganda Demographic Health Survey (UDHS) data focussing on child health, vaccination status and maternal education (51, 52), and data on Indigenous communities are scarce. Our mixed-methods design allowed to explore the socio-cultural and environmental barriers to child feeding practices among the Indigenous Batwa population for which there are no data on the UDHS. Also, we compared the information with the neighbouring Bakiga who shared a similar experience in terms of breastfeeding and complementary feeding pratices.’* |
| 7) Remove the spacing after pages 6 and 23 On line 535 space the words "likbetween" On line 48 put a comma after 2019 Lable tables in a standard manner throughout text instead of Tab write in full Table | We addressed all minor comments raised by the reviewer in the text. |
| **Reference list** | **Author comments/response** |
| 1) Please review your reference list to ensure that it is complete and correct. If you have cited papers that have been retracted, please include the rationale for doing so in the manuscript text, or remove these references and replace them with relevant current references. Any changes to the reference list should be mentioned in the rebuttal letter that accompanies your revised manuscript. If you need to cite a retracted article, indicate the article’s retracted status in the References list and also include a citation and full reference for the retraction notice. | The references list have been updated. Some new references have been added (ref 72,73,75) and we have updated the following references:  Ref. 29: previous reference 29 removed as it was an error;  Ref 30: previous reference 30 removed as it was an error;  Ref. 34: previous reference 34 changed with published literature (reference 24 and 34);  Ref.41: previous reference 41, now 39, has been updated as the article was published. |
